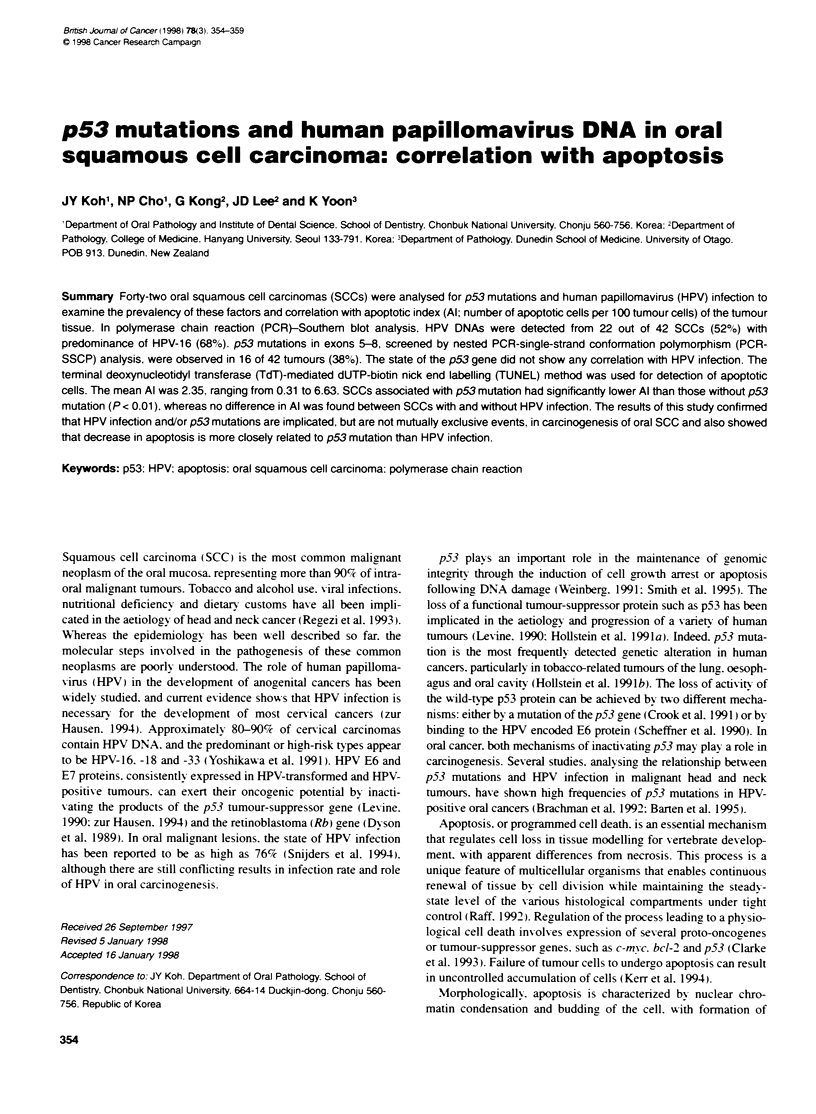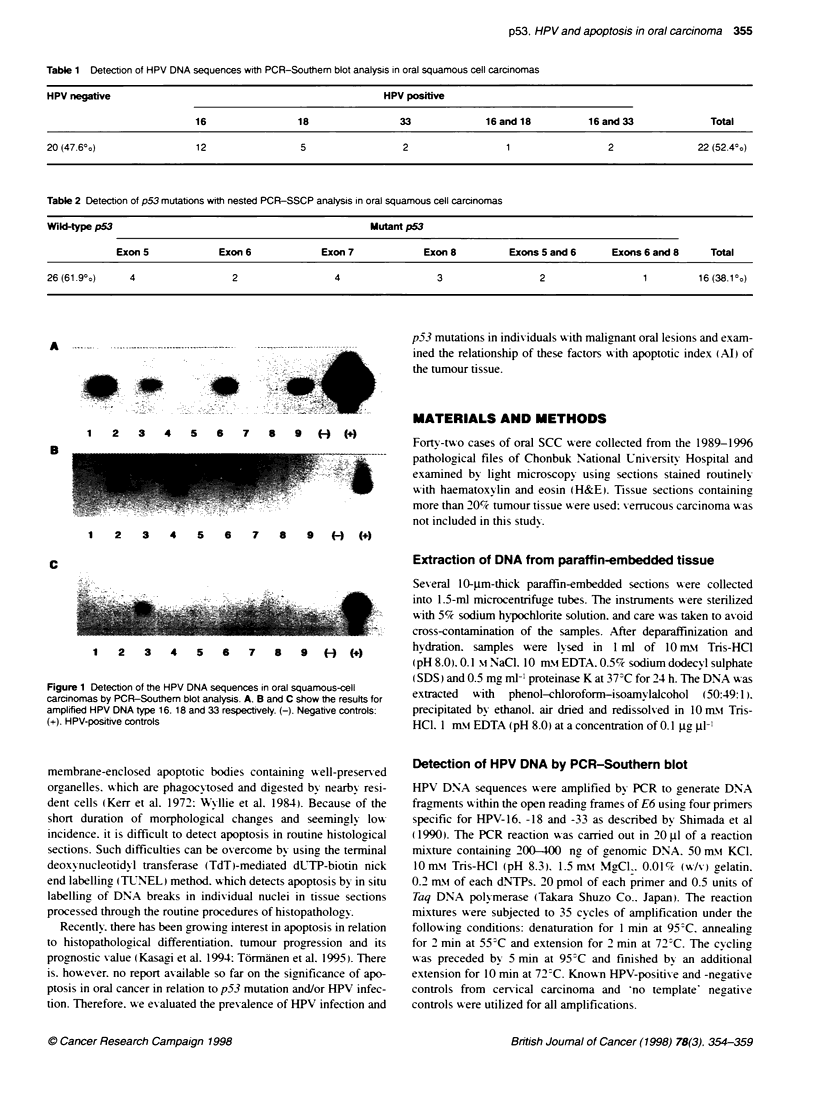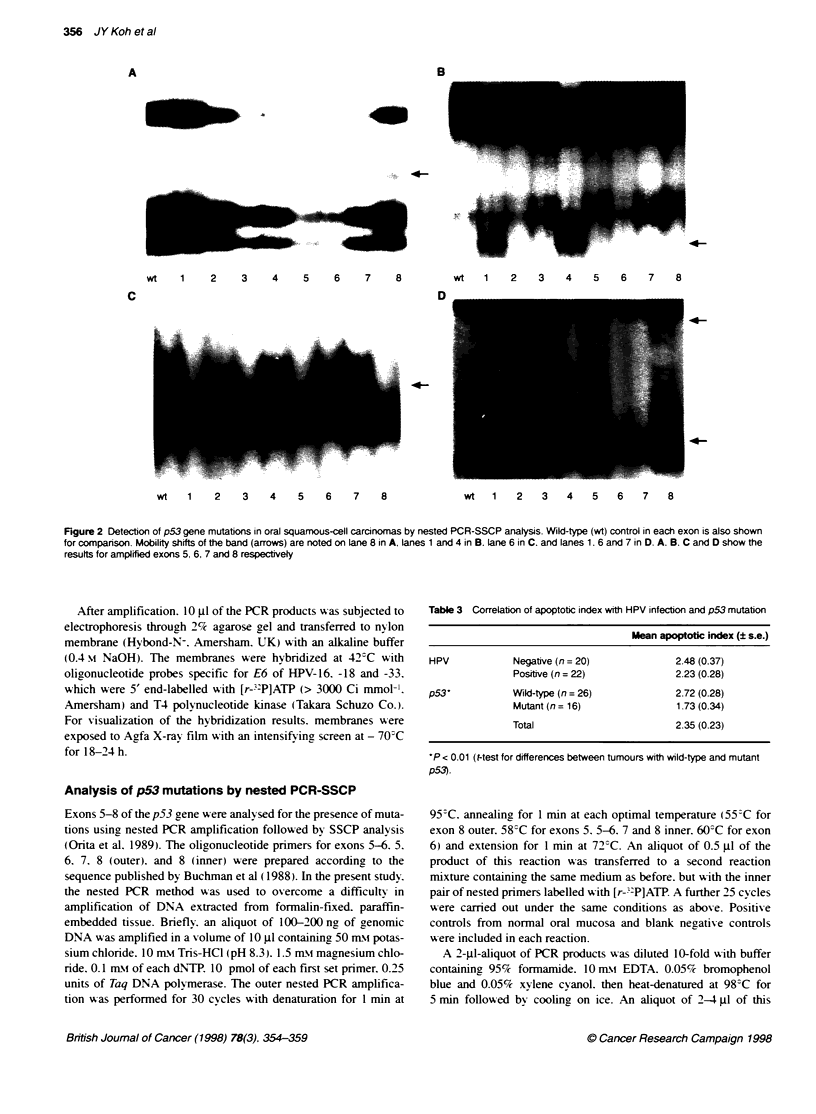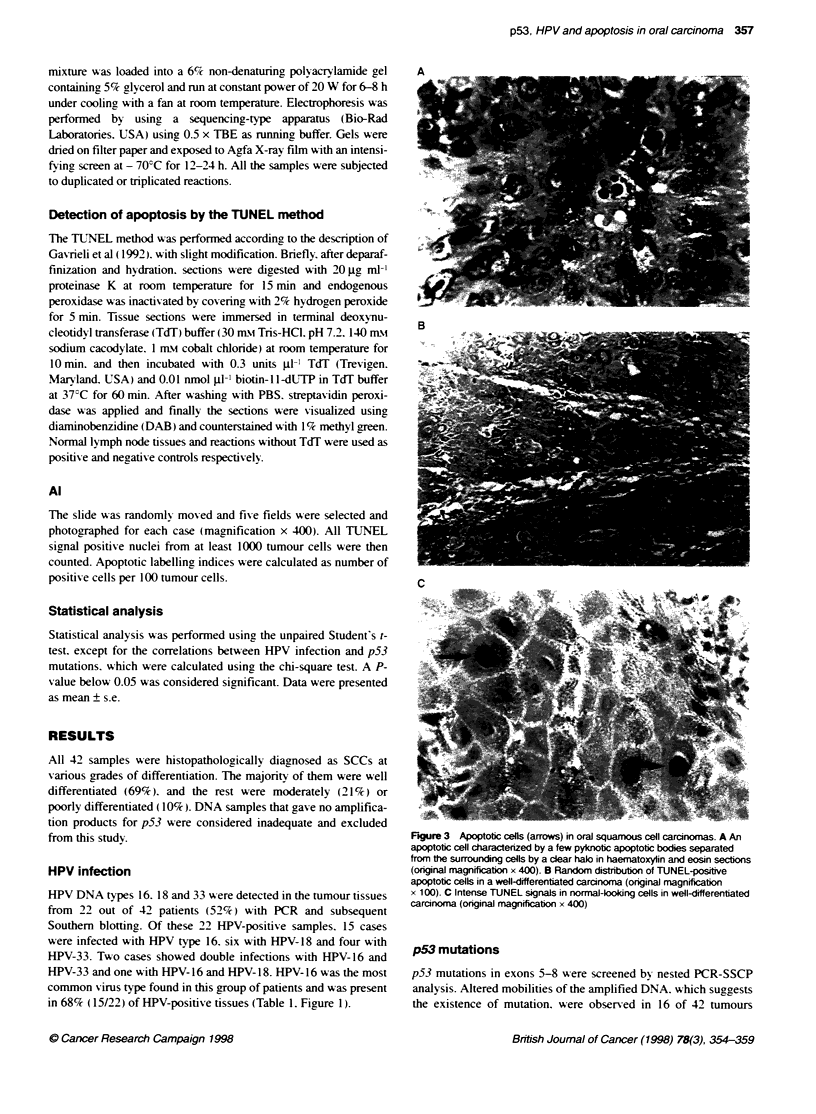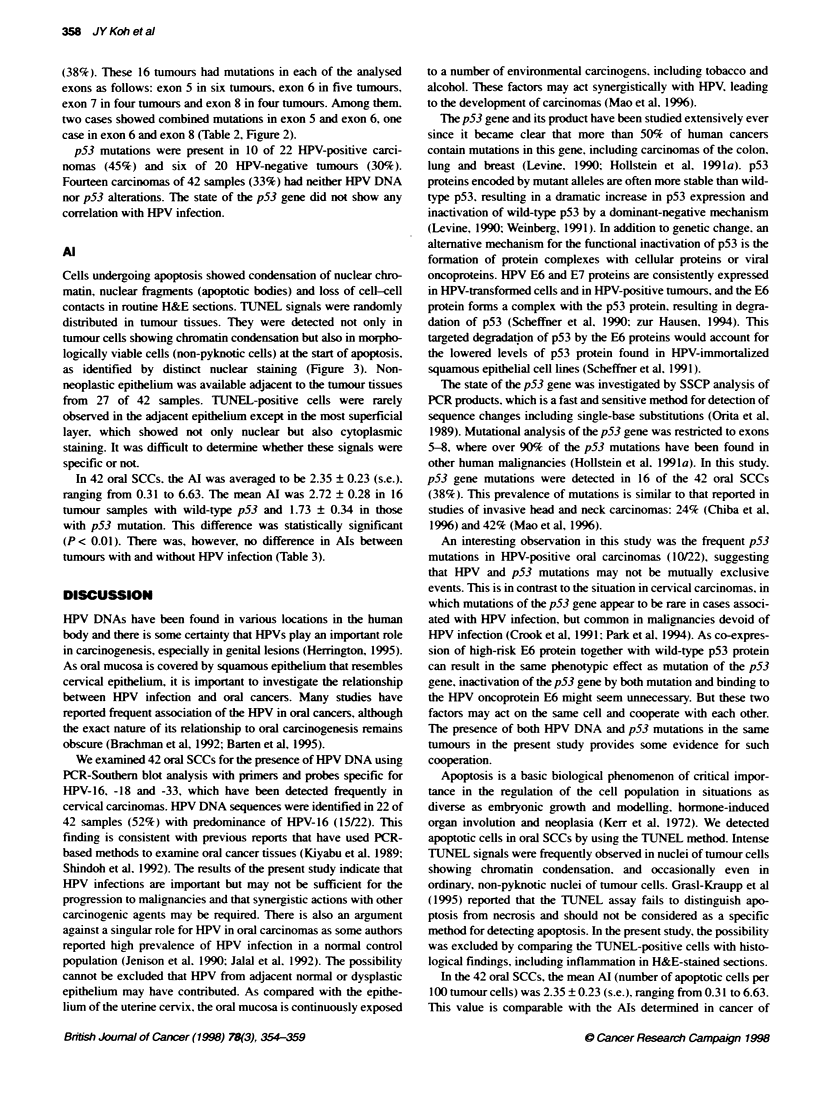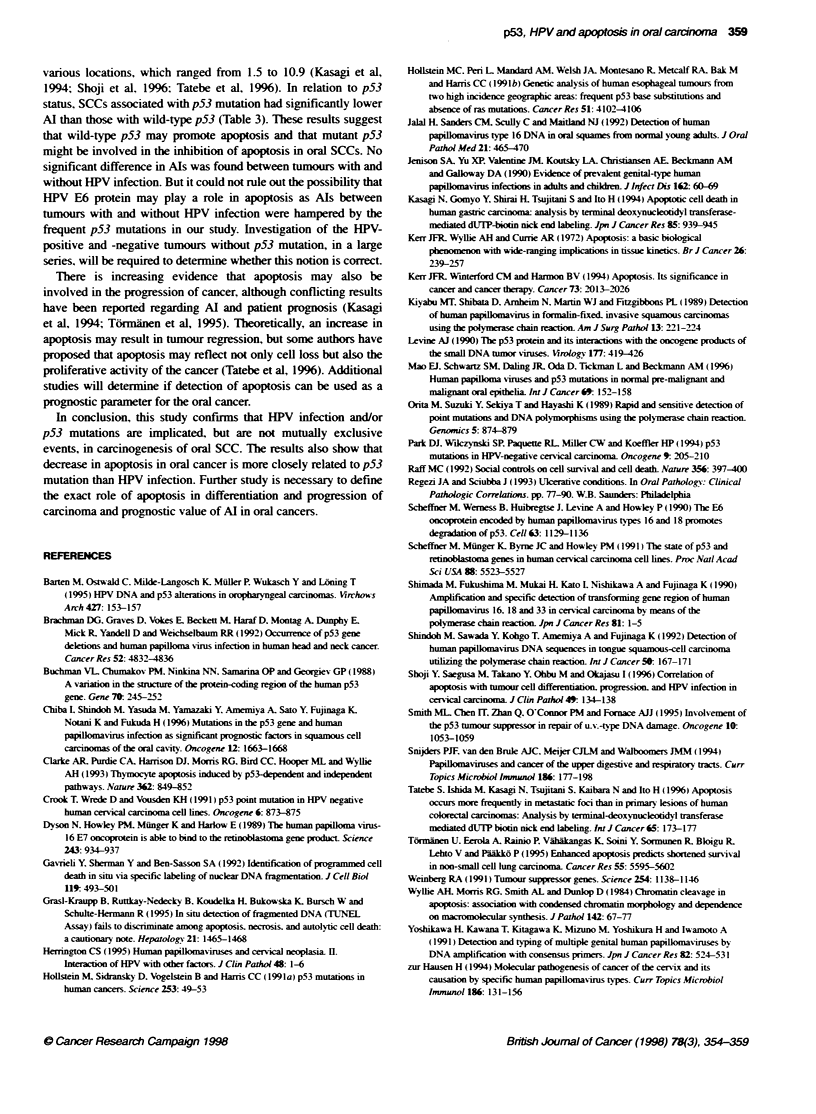# p53 mutations and human papillomavirus DNA in oral squamous cell carcinoma: correlation with apoptosis.

**DOI:** 10.1038/bjc.1998.498

**Published:** 1998-08

**Authors:** J. Y. Koh, N. P. Cho, G. Kong, J. D. Lee, K. Yoon

**Affiliations:** Department of Oral Pathology and Institute of Dental Science, School of Dentistry, Chonbuk National University, Chonju, Korea.

## Abstract

**Images:**


British Journal of Cancer (1998) 78(3), 354–359
© 1998 Cancer Research Campaign

### *p53* mutations and human papillomavirus DNA in oral squamous cell carcinoma: correlation with apoptosis

**JY Koh[1], NP Cho[1], G Kong[2], JD Lee[2] and K Yoon[3]**

[1]Department of Oral Pathology and Institute of Dental Science, School of Dentistry, Chonbuk National University, Chonju 560-756, Korea; [2]Department of Pathology, College of Medicine, Hanyang University, Seoul 133-791, Korea; [3]Department of Pathology, Dunedin School of Medicine, University of Otago, POB 913, Dunedin, New Zealand

**Summary** Forty-two oral squamous cell carcinomas (SCCs) were analysed for *p53* mutations and human papillomavirus (HPV) infection to examine the prevalency of these factors and correlation with apoptotic index (AI; number of apoptotic cells per 100 tumour cells) of the tumour tissue. In polymerase chain reaction (PCR)–Southern blot analysis, HPV DNAs were detected from 22 out of 42 SCCs (52%) with predominance of HPV-16 (68%). *p53* mutations in exons 5–8, screened by nested PCR-single-strand conformation polymorphism (PCR-SSCP) analysis, were observed in 16 of 42 tumours (38%). The state of the *p53* gene did not show any correlation with HPV infection. The terminal deoxynucleotidyl transferase (TdT)-mediated dUTP-biotin nick end labelling (TUNEL) method was used for detection of apoptotic cells. The mean AI was 2.35, ranging from 0.31 to 6.63. SCCs associated with *p53* mutation had significantly lower AI than those without *p53* mutation ($P < 0.01$), whereas no difference in AI was found between SCCs with and without HPV infection. The results of this study confirmed that HPV infection and/or *p53* mutations are implicated, but are not mutually exclusive events, in carcinogenesis of oral SCC and also showed that decrease in apoptosis is more closely related to *p53* mutation than HPV infection.

**Keywords:** p53; HPV; apoptosis; oral squamous cell carcinoma; polymerase chain reaction

Squamous cell carcinoma (SCC) is the most common malignant neoplasm of the oral mucosa, representing more than 90% of intra-oral malignant tumours. Tobacco and alcohol use, viral infections, nutritional deficiency and dietary customs have all been implicated in the aetiology of head and neck cancer (Regezi et al, 1993). Whereas the epidemiology has been well described so far, the molecular steps involved in the pathogenesis of these common neoplasms are poorly understood. The role of human papillomavirus (HPV) in the development of anogenital cancers has been widely studied, and current evidence shows that HPV infection is necessary for the development of most cervical cancers (zur Hausen, 1994). Approximately 80–90% of cervical carcinomas contain HPV DNA, and the predominant or high-risk types appear to be HPV-16, -18 and -33 (Yoshikawa et al, 1991). HPV E6 and E7 proteins, consistently expressed in HPV-transformed and HPV-positive tumours, can exert their oncogenic potential by inactivating the products of the *p53* tumour-suppressor gene (Levine, 1990; zur Hausen, 1994) and the retinoblastoma (*Rb*) gene (Dyson et al, 1989). In oral malignant lesions, the state of HPV infection has been reported to be as high as 76% (Snijders et al, 1994), although there are still conflicting results in infection rate and role of HPV in oral carcinogenesis.

*Received 26 September 1997*
*Revised 5 January 1998*
*Accepted 16 January 1998*

*Correspondence to:* JY Koh, Department of Oral Pathology, School of Dentistry, Chonbuk National University, 664-14 Duckjin-dong, Chonju 560-756, Republic of Korea

*p53* plays an important role in the maintenance of genomic integrity through the induction of cell growth arrest or apoptosis following DNA damage (Weinberg, 1991; Smith et al, 1995). The loss of a functional tumour-suppressor protein such as p53 has been implicated in the aetiology and progression of a variety of human tumours (Levine, 1990; Hollstein et al, 1991*a*). Indeed, *p53* mutation is the most frequently detected genetic alteration in human cancers, particularly in tobacco-related tumours of the lung, oesophagus and oral cavity (Hollstein et al, 1991*b*). The loss of activity of the wild-type p53 protein can be achieved by two different mechanisms: either by a mutation of the *p53* gene (Crook et al, 1991) or by binding to the HPV encoded E6 protein (Scheffner et al, 1990). In oral cancer, both mechanisms of inactivating *p53* may play a role in carcinogenesis. Several studies, analysing the relationship between *p53* mutations and HPV infection in malignant head and neck tumours, have shown high frequencies of *p53* mutations in HPV-positive oral cancers (Brachman et al, 1992; Barten et al, 1995).

Apoptosis, or programmed cell death, is an essential mechanism that regulates cell loss in tissue modelling for vertebrate development, with apparent differences from necrosis. This process is a unique feature of multicellular organisms that enables continuous renewal of tissue by cell division while maintaining the steady-state level of the various histological compartments under tight control (Raff, 1992). Regulation of the process leading to a physiological cell death involves expression of several proto-oncogenes or tumour-suppressor genes, such as *c-myc*, *bcl-2* and *p53* (Clarke et al, 1993). Failure of tumour cells to undergo apoptosis can result in uncontrolled accumulation of cells (Kerr et al, 1994).

Morphologically, apoptosis is characterized by nuclear chromatin condensation and budding of the cell, with formation of

**Table 1** Detection of HPV DNA sequences with PCR–Southern blot analysis in oral squamous cell carcinomas

| HPV negative | HPV positive | | | | | |
|---|---|---|---|---|---|---|
| | 16 | 18 | 33 | 16 and 18 | 16 and 33 | Total |
| 20 (47.6%) | 12 | 5 | 2 | 1 | 2 | 22 (52.4%) |

**Table 2** Detection of *p53* mutations with nested PCR–SSCP analysis in oral squamous cell carcinomas

| Wild-type *p53* | Mutant *p53* | | | | | | |
|---|---|---|---|---|---|---|---|
| | Exon 5 | Exon 6 | Exon 7 | Exon 8 | Exons 5 and 6 | Exons 6 and 8 | Total |
| 26 (61.9%) | 4 | 2 | 4 | 3 | 2 | 1 | 16 (38.1%) |

**Figure 1** Detection of the HPV DNA sequences in oral squamous-cell carcinomas by PCR–Southern blot analysis. **A**, **B** and **C** show the results for amplified HPV DNA type 16, 18 and 33 respectively. (–), Negative controls; (+), HPV-positive controls membrane-enclosed apoptotic bodies containing well-preserved organelles, which are phagocytosed and digested by nearby resident cells (Kerr et al, 1972; Wyllie et al, 1984). Because of the short duration of morphological changes and seemingly low incidence, it is difficult to detect apoptosis in routine histological sections. Such difficulties can be overcome by using the terminal deoxynucleotidyl transferase (TdT)-mediated dUTP-biotin nick end labelling (TUNEL) method, which detects apoptosis by in situ labelling of DNA breaks in individual nuclei in tissue sections processed through the routine procedures of histopathology.

Recently, there has been growing interest in apoptosis in relation to histopathological differentiation, tumour progression and its prognostic value (Kasagi et al, 1994; Törmänen et al, 1995). There is, however, no report available so far on the significance of apoptosis in oral cancer in relation to *p53* mutation and/or HPV infection. Therefore, we evaluated the prevalence of HPV infection and *p53* mutations in individuals with malignant oral lesions and examined the relationship of these factors with apoptotic index (AI) of the tumour tissue.

### MATERIALS AND METHODS

Forty-two cases of oral SCC were collected from the 1989–1996 pathological files of Chonbuk National University Hospital and examined by light microscopy using sections stained routinely with haematoxylin and eosin (H&E). Tissue sections containing more than 20% tumour tissue were used; verrucous carcinoma was not included in this study.

### Extraction of DNA from paraffin-embedded tissue

Several 10-μm-thick paraffin-embedded sections were collected into 1.5-ml microcentrifuge tubes. The instruments were sterilized with 5% sodium hypochlorite solution, and care was taken to avoid cross-contamination of the samples. After deparaffinization and hydration, samples were lysed in 1 ml of 10 mM Tris-HCl (pH 8.0), 0.1 M NaCl, 10 mM EDTA, 0.5% sodium dodecyl sulphate (SDS) and 0.5 mg ml$^{-1}$ proteinase K at 37°C for 24 h. The DNA was extracted with phenol–chloroform–isoamylalcohol (50:49:1), precipitated by ethanol, air dried and redissolved in 10 mM Tris-HCl, 1 mM EDTA (pH 8.0) at a concentration of 0.1 μg μl$^{-1}$

### Detection of HPV DNA by PCR–Southern blot

HPV DNA sequences were amplified by PCR to generate DNA fragments within the open reading frames of *E6* using four primers specific for HPV-16, -18 and -33 as described by Shimada et al (1990). The PCR reaction was carried out in 20 μl of a reaction mixture containing 200–400 ng of genomic DNA, 50 mM KCl, 10 mM Tris-HCl (pH 8.3), 1.5 mM $MgCl_2$, 0.01% (w/v) gelatin, 0.2 mM of each dNTPs, 20 pmol of each primer and 0.5 units of *Taq* DNA polymerase (Takara Shuzo Co., Japan). The reaction mixtures were subjected to 35 cycles of amplification under the following conditions: denaturation for 1 min at 95°C, annealing for 2 min at 55°C and extension for 2 min at 72°C. The cycling was preceded by 5 min at 95°C and finished by an additional extension for 10 min at 72°C. Known HPV-positive and -negative controls from cervical carcinoma and 'no template' negative controls were utilized for all amplifications.

© Cancer Research Campaign 1998

**Figure 2** Detection of *p53* gene mutations in oral squamous-cell carcinomas by nested PCR-SSCP analysis. Wild-type (wt) control in each exon is also shown for comparison. Mobility shifts of the band (arrows) are noted on lane 8 in **A**, lanes 1 and 4 in **B**, lane 6 in **C**, and lanes 1, 6 and 7 in **D**. **A**, **B**, **C** and **D** show the results for amplified exons 5, 6, 7 and 8 respectively

After amplification, 10 µl of the PCR products was subjected to electrophoresis through 2% agarose gel and transferred to nylon membrane (Hybond-N$^{+}$, Amersham, UK) with an alkaline buffer (0.4 M NaOH). The membranes were hybridized at 42°C with oligonucleotide probes specific for *E6* of HPV-16, -18 and -33, which were 5′ end-labelled with [γ-$^{32}$P]ATP (> 3000 Ci $mmol^{-1}$, Amersham) and T4 polynucleotide kinase (Takara Schuzo Co.). For visualization of the hybridization results, membranes were exposed to Agfa X-ray film with an intensifying screen at – 70°C for 18–24 h.

**Table 3** Correlation of apoptotic index with HPV infection and *p53* mutation

| | | Mean apoptotic index (± s.e.) |
|---|---|---|
| HPV | Negative (*n* = 20) | 2.48 (0.37) |
| | Positive (*n* = 22) | 2.23 (0.28) |
| *p53** | Wild-type (*n* = 26) | 2.72 (0.28) |
| | Mutant (*n* = 16) | 1.73 (0.34) |
| | Total | 2.35 (0.23) |

**P* < 0.01 (*t*-test for differences between tumours with wild-type and mutant *p53*).

### Analysis of *p53* mutations by nested PCR-SSCP

Exons 5–8 of the *p53* gene were analysed for the presence of mutations using nested PCR amplification followed by SSCP analysis (Orita et al, 1989). The oligonucleotide primers for exons 5–6, 5, 6, 7, 8 (outer), and 8 (inner) were prepared according to the sequence published by Buchman et al (1988). In the present study, the nested PCR method was used to overcome a difficulty in amplification of DNA extracted from formalin-fixed, paraffin-embedded tissue. Briefly, an aliquot of 100–200 ng of genomic DNA was amplified in a volume of 10 µl containing 50 mM potassium chloride, 10 mM Tris-HCl (pH 8.3), 1.5 mM magnesium chloride, 0.1 mM of each dNTP, 10 pmol of each first set primer, 0.25 units of *Taq* DNA polymerase. The outer nested PCR amplification was performed for 30 cycles with denaturation for 1 min at 95°C, annealing for 1 min at each optimal temperature (55°C for exon 8 outer, 58°C for exons 5, 5–6, 7 and 8 inner, 60°C for exon 6) and extension for 1 min at 72°C. An aliquot of 0.5 µl of the product of this reaction was transferred to a second reaction mixture containing the same medium as before, but with the inner pair of nested primers labelled with [γ-$^{32}$P]ATP. A further 25 cycles were carried out under the same conditions as above. Positive controls from normal oral mucosa and blank negative controls were included in each reaction.

A 2-µl-aliquot of PCR products was diluted 10-fold with buffer containing 95% formamide, 10 mM EDTA, 0.05% bromophenol blue and 0.05% xylene cyanol, then heat-denatured at 98°C for 5 min followed by cooling on ice. An aliquot of 2–4 µl of this

© Cancer Research Campaign 1998

mixture was loaded into a 6% non-denaturing polyacrylamide gel containing 5% glycerol and run at constant power of 20 W for 6–8 h under cooling with a fan at room temperature. Electrophoresis was performed by using a sequencing-type apparatus (Bio-Rad Laboratories, USA) using 0.5 × TBE as running buffer. Gels were dried on filter paper and exposed to Agfa X-ray film with an intensifying screen at – 70°C for 12–24 h. All the samples were subjected to duplicated or triplicated reactions.

### Detection of apoptosis by the TUNEL method

The TUNEL method was performed according to the description of Gavrieli et al (1992), with slight modification. Briefly, after deparaffinization and hydration, sections were digested with 20 µg $ml^{-1}$ proteinase K at room temperature for 15 min and endogenous peroxidase was inactivated by covering with 2% hydrogen peroxide for 5 min. Tissue sections were immersed in terminal deoxynucleotidyl transferase (TdT) buffer (30 mM Tris-HCl, pH 7.2, 140 mM sodium cacodylate, 1 mM cobalt chloride) at room temperature for 10 min, and then incubated with 0.3 units $\mu l^{-1}$ TdT (Trevigen, Maryland, USA) and 0.01 nmol $\mu l^{-1}$ biotin-11-dUTP in TdT buffer at 37°C for 60 min. After washing with PBS, streptavidin peroxidase was applied and finally the sections were visualized using diaminobenzidine (DAB) and counterstained with 1% methyl green. Normal lymph node tissues and reactions without TdT were used as positive and negative controls respectively.

### AI

The slide was randomly moved and five fields were selected and photographed for each case (magnification × 400). All TUNEL signal positive nuclei from at least 1000 tumour cells were then counted. Apoptotic labelling indices were calculated as number of positive cells per 100 tumour cells.

### Statistical analysis

Statistical analysis was performed using the unpaired Student's *t*-test, except for the correlations between HPV infection and *p53* mutations, which were calculated using the chi-square test. A *P*-value below 0.05 was considered significant. Data were presented as mean ± s.e.

### RESULTS

All 42 samples were histopathologically diagnosed as SCCs at various grades of differentiation. The majority of them were well differentiated (69%), and the rest were moderately (21%) or poorly differentiated (10%). DNA samples that gave no amplification products for *p53* were considered inadequate and excluded from this study.

### HPV infection

HPV DNA types 16, 18 and 33 were detected in the tumour tissues from 22 out of 42 patients (52%) with PCR and subsequent Southern blotting. Of these 22 HPV-positive samples, 15 cases were infected with HPV type 16, six with HPV-18 and four with HPV-33. Two cases showed double infections with HPV-16 and HPV-33 and one with HPV-16 and HPV-18. HPV-16 was the most common virus type found in this group of patients and was present in 68% (15/22) of HPV-positive tissues (Table 1, Figure 1).

**Figure 3** Apoptotic cells (arrows) in oral squamous cell carcinomas. **A** An apoptotic cell characterized by a few pyknotic apoptotic bodies separated from the surrounding cells by a clear halo in haematoxylin and eosin sections (original magnification × 400). **B** Random distribution of TUNEL-positive apoptotic cells in a well-differentiated carcinoma (original magnification × 100). **C** Intense TUNEL signals in normal-looking cells in well-differentiated carcinoma (original magnification × 400)

### *p53* mutations

*p53* mutations in exons 5–8 were screened by nested PCR-SSCP analysis. Altered mobilities of the amplified DNA, which suggests the existence of mutation, were observed in 16 of 42 tumours

© Cancer Research Campaign 1998

(38%). These 16 tumours had mutations in each of the analysed exons as follows: exon 5 in six tumours, exon 6 in five tumours, exon 7 in four tumours and exon 8 in four tumours. Among them, two cases showed combined mutations in exon 5 and exon 6, one case in exon 6 and exon 8 (Table 2, Figure 2).

*p53* mutations were present in 10 of 22 HPV-positive carcinomas (45%) and six of 20 HPV-negative tumours (30%). Fourteen carcinomas of 42 samples (33%) had neither HPV DNA nor *p53* alterations. The state of the *p53* gene did not show any correlation with HPV infection.

### AI

Cells undergoing apoptosis showed condensation of nuclear chromatin, nuclear fragments (apoptotic bodies) and loss of cell–cell contacts in routine H&E sections. TUNEL signals were randomly distributed in tumour tissues. They were detected not only in tumour cells showing chromatin condensation but also in morphologically viable cells (non-pyknotic cells) at the start of apoptosis, as identified by distinct nuclear staining (Figure 3). Non-neoplastic epithelium was available adjacent to the tumour tissues from 27 of 42 samples. TUNEL-positive cells were rarely observed in the adjacent epithelium except in the most superficial layer, which showed not only nuclear but also cytoplasmic staining. It was difficult to determine whether these signals were specific or not.

In 42 oral SCCs, the AI was averaged to be 2.35 ± 0.23 (s.e.), ranging from 0.31 to 6.63. The mean AI was 2.72 ± 0.28 in 16 tumour samples with wild-type *p53* and 1.73 ± 0.34 in those with *p53* mutation. This difference was statistically significant ($P < 0.01$). There was, however, no difference in AIs between tumours with and without HPV infection (Table 3).

### DISCUSSION

HPV DNAs have been found in various locations in the human body and there is some certainty that HPVs play an important role in carcinogenesis, especially in genital lesions (Herrington, 1995). As oral mucosa is covered by squamous epithelium that resembles cervical epithelium, it is important to investigate the relationship between HPV infection and oral cancers. Many studies have reported frequent association of the HPV in oral cancers, although the exact nature of its relationship to oral carcinogenesis remains obscure (Brachman et al, 1992; Barten et al, 1995).

We examined 42 oral SCCs for the presence of HPV DNA using PCR-Southern blot analysis with primers and probes specific for HPV-16, -18 and -33, which have been detected frequently in cervical carcinomas. HPV DNA sequences were identified in 22 of 42 samples (52%) with predominance of HPV-16 (15/22). This finding is consistent with previous reports that have used PCR-based methods to examine oral cancer tissues (Kiyabu et al. 1989; Shindoh et al, 1992). The results of the present study indicate that HPV infections are important but may not be sufficient for the progression to malignancies and that synergistic actions with other carcinogenic agents may be required. There is also an argument against a singular role for HPV in oral carcinomas as some authors reported high prevalence of HPV infection in a normal control population (Jenison et al, 1990; Jalal et al, 1992). The possibility cannot be excluded that HPV from adjacent normal or dysplastic epithelium may have contributed. As compared with the epithelium of the uterine cervix, the oral mucosa is continuously exposed to a number of environmental carcinogens, including tobacco and alcohol. These factors may act synergistically with HPV, leading to the development of carcinomas (Mao et al, 1996).

The *p53* gene and its product have been studied extensively ever since it became clear that more than 50% of human cancers contain mutations in this gene, including carcinomas of the colon, lung and breast (Levine, 1990; Hollstein et al, 1991*a*). p53 proteins encoded by mutant alleles are often more stable than wild-type p53, resulting in a dramatic increase in p53 expression and inactivation of wild-type p53 by a dominant-negative mechanism (Levine, 1990; Weinberg, 1991). In addition to genetic change, an alternative mechanism for the functional inactivation of p53 is the formation of protein complexes with cellular proteins or viral oncoproteins. HPV E6 and E7 proteins are consistently expressed in HPV-transformed cells and in HPV-positive tumours, and the E6 protein forms a complex with the p53 protein, resulting in degradation of p53 (Scheffner et al, 1990; zur Hausen, 1994). This targeted degradation of p53 by the E6 proteins would account for the lowered levels of p53 protein found in HPV-immortalized squamous epithelial cell lines (Scheffner et al, 1991).

The state of the *p53* gene was investigated by SSCP analysis of PCR products, which is a fast and sensitive method for detection of sequence changes including single-base substitutions (Orita et al, 1989). Mutational analysis of the *p53* gene was restricted to exons 5–8, where over 90% of the *p53* mutations have been found in other human malignancies (Hollstein et al, 1991*a*). In this study, *p53* gene mutations were detected in 16 of the 42 oral SCCs (38%). This prevalence of mutations is similar to that reported in studies of invasive head and neck carcinomas: 24% (Chiba et al, 1996) and 42% (Mao et al, 1996).

An interesting observation in this study was the frequent *p53* mutations in HPV-positive oral carcinomas (10/22), suggesting that HPV and *p53* mutations may not be mutually exclusive events. This is in contrast to the situation in cervical carcinomas, in which mutations of the *p53* gene appear to be rare in cases associated with HPV infection, but common in malignancies devoid of HPV infection (Crook et al, 1991; Park et al, 1994). As co-expression of high-risk E6 protein together with wild-type p53 protein can result in the same phenotypic effect as mutation of the *p53* gene, inactivation of the *p53* gene by both mutation and binding to the HPV oncoprotein E6 might seem unnecessary. But these two factors may act on the same cell and cooperate with each other. The presence of both HPV DNA and *p53* mutations in the same tumours in the present study provides some evidence for such cooperation.

Apoptosis is a basic biological phenomenon of critical importance in the regulation of the cell population in situations as diverse as embryonic growth and modelling, hormone-induced organ involution and neoplasia (Kerr et al, 1972). We detected apoptotic cells in oral SCCs by using the TUNEL method. Intense TUNEL signals were frequently observed in nuclei of tumour cells showing chromatin condensation, and occasionally even in ordinary, non-pyknotic nuclei of tumour cells. Grasl-Kraupp et al (1995) reported that the TUNEL assay fails to distinguish apoptosis from necrosis and should not be considered as a specific method for detecting apoptosis. In the present study, the possibility was excluded by comparing the TUNEL-positive cells with histological findings, including inflammation in H&E-stained sections.

In the 42 oral SCCs, the mean AI (number of apoptotic cells per 100 tumour cells) was 2.35 ± 0.23 (s.e.), ranging from 0.31 to 6.63. This value is comparable with the AIs determined in cancer of

 © Cancer Research Campaign 1998

various locations, which ranged from 1.5 to 10.9 (Kasagi et al, 1994; Shoji et al, 1996; Tatebe et al, 1996). In relation to *p53* status, SCCs associated with *p53* mutation had significantly lower AI than those with wild-type *p53* (Table 3). These results suggest that wild-type *p53* may promote apoptosis and that mutant *p53* might be involved in the inhibition of apoptosis in oral SCCs. No significant difference in AIs was found between tumours with and without HPV infection. But it could not rule out the possibility that HPV E6 protein may play a role in apoptosis as AIs between tumours with and without HPV infection were hampered by the frequent *p53* mutations in our study. Investigation of the HPV-positive and -negative tumours without *p53* mutation, in a large series, will be required to determine whether this notion is correct.

There is increasing evidence that apoptosis may also be involved in the progression of cancer, although conflicting results have been reported regarding AI and patient prognosis (Kasagi et al, 1994; Törmänen et al, 1995). Theoretically, an increase in apoptosis may result in tumour regression, but some authors have proposed that apoptosis may reflect not only cell loss but also the proliferative activity of the cancer (Tatebe et al, 1996). Additional studies will determine if detection of apoptosis can be used as a prognostic parameter for the oral cancer.

In conclusion, this study confirms that HPV infection and/or *p53* mutations are implicated, but are not mutually exclusive events, in carcinogenesis of oral SCC. The results also show that decrease in apoptosis in oral cancer is more closely related to *p53* mutation than HPV infection. Further study is necessary to define the exact role of apoptosis in differentiation and progression of carcinoma and prognostic value of AI in oral cancers.

### REFERENCES

Barten M, Ostwald C, Milde-Langosch K, Müller P, Wukasch Y and Löning T (1995) HPV DNA and p53 alterations in oropharyngeal carcinomas. *Virchows Arch* **427**: 153–157

Brachman DG, Graves D, Vokes E, Beckett M, Haraf D, Montag A, Dunphy E, Mick R, Yandell D and Weichselbaum RR (1992) Occurrence of p53 gene deletions and human papilloma virus infection in human head and neck cancer. *Cancer Res* **52**: 4832–4836

Buchman VL, Chumakov PM, Ninkina NN, Samarina OP and Georgiev GP (1988) A variation in the structure of the protein-coding region of the human p53 gene. *Gene* **70**: 245–252

Chiba I, Shindoh M, Yasuda M, Yamazaki Y, Amemiya A, Sato Y, Fujinaga K, Notani K and Fukuda H (1996) Mutations in the p53 gene and human papillomavirus infection as significant prognostic factors in squamous cell carcinomas of the oral cavity. *Oncogene* **12**: 1663–1668

Clarke AR, Purdie CA, Harrison DJ, Morris RG, Bird CC, Hooper ML and Wyllie AH (1993) Thymocyte apoptosis induced by p53-dependent and independent pathways. *Nature* **362**: 849–852

Crook T, Wrede D and Vousden KH (1991) p53 point mutation in HPV negative human cervical carcinoma cell lines. *Oncogene* **6**: 873–875

Dyson N, Howley PM, Münger K and Harlow E (1989) The human papilloma virus-16 E7 oncoprotein is able to bind to the retinoblastoma gene product. *Science* **243**: 934–937

Gavrieli Y, Sherman Y and Ben-Sasson SA (1992) Identification of programmed cell death in situ via specific labeling of nuclear DNA fragmentation. *J Cell Biol* **119**: 493–501

Grasl-Kraupp B, Ruttkay-Nedecky B, Koudelka H, Bukowska K, Bursch W and Schulte-Hermann R (1995) In situ detection of fragmented DNA (TUNEL Assay) fails to discriminate among apoptosis, necrosis, and autolytic cell death: a cautionary note. *Hepatology* **21**: 1465–1468

Herrington CS (1995) Human papillomaviruses and cervical neoplasia. II. Interaction of HPV with other factors. *J Clin Pathol* **48**: 1–6

Hollstein M, Sidransky D, Vogelstein B and Harris CC (1991*a*) p53 mutations in human cancers. *Science* **253**: 49–53

Hollstein MC, Peri L, Mandard AM, Welsh JA, Montesano R, Metcalf RA, Bak M and Harris CC (1991*b*) Genetic analysis of human esophageal tumours from two high incidence geographic areas: frequent p53 base substitutions and absence of ras mutations. *Cancer Res* **51**: 4102–4106

Jalal H, Sanders CM, Scully C and Maitland NJ (1992) Detection of human papillomavirus type 16 DNA in oral squames from normal young adults. *J Oral Pathol Med* **21**: 465–470

Jenison SA, Yu XP, Valentine JM, Koutsky LA, Christiansen AE, Beckmann AM and Galloway DA (1990) Evidence of prevalent genital-type human papillomavirus infections in adults and children. *J Infect Dis* **162**: 60–69

Kasagi N, Gomyo Y, Shirai H, Tsujitani S and Ito H (1994) Apoptotic cell death in human gastric carcinoma: analysis by terminal deoxynucleotidyl transferase-mediated dUTP-biotin nick end labeling. *Jpn J Cancer Res* **85**: 939–945

Kerr JFR, Wyllie AH and Currie AR (1972) Apoptosis: a basic biological phenomenon with wide-ranging implications in tissue kinetics. *Br J Cancer* **26**: 239–257

Kerr JFR, Winterford CM and Harmon BV (1994) Apoptosis. Its significance in cancer and cancer therapy. *Cancer* **73**: 2013–2026

Kiyabu MT, Shibata D, Arnheim N, Martin WJ and Fitzgibbons PL (1989) Detection of human papillomavirus in formalin-fixed, invasive squamous carcinomas using the polymerase chain reaction. *Am J Surg Pathol* **13**: 221–224

Levine AJ (1990) The p53 protein and its interactions with the oncogene products of the small DNA tumor viruses. *Virology* **177**: 419–426

Mao EJ, Schwartz SM, Daling JR, Oda D, Tickman L and Beckmann AM (1996) Human papilloma viruses and p53 mutations in normal pre-malignant and malignant oral epithelia. *Int J Cancer* **69**: 152–158

Orita M, Suzuki Y, Sekiya T and Hayashi K (1989) Rapid and sensitive detection of point mutations and DNA polymorphisms using the polymerase chain reaction. *Genomics* **5**: 874–879

Park DJ, Wilczynski SP, Paquette RL, Miller CW and Koeffler HP (1994) p53 mutations in HPV-negative cervical carcinoma. *Oncogene* **9**: 205–210

Raff MC (1992) Social controls on cell survival and cell death. *Nature* **356**: 397–400

Regezi JA and Sciubba J (1993) Ulcerative conditions. In *Oral Pathology: Clinical Pathologic Correlations*. pp. 77–90. W.B. Saunders: Philadelphia

Scheffner M, Werness B, Huibregtse J, Levine A and Howley P (1990) The E6 oncoprotein encoded by human papillomavirus types 16 and 18 promotes degradation of p53. *Cell* **63**: 1129–1136

Scheffner M, Münger K, Byrne JC and Howley PM (1991) The state of p53 and retinoblastoma genes in human cervical carcinoma cell lines. *Proc Natl Acad Sci USA* **88**: 5523–5527

Shimada M, Fukushima M, Mukai H, Kato I, Nishikawa A and Fujinaga K (1990) Amplification and specific detection of transforming gene region of human papillomavirus 16, 18 and 33 in cervical carcinoma by means of the polymerase chain reaction. *Jpn J Cancer Res* **81**: 1–5

Shindoh M, Sawada Y, Kohgo T, Amemiya A and Fujinaga K (1992) Detection of human papillomavirus DNA sequences in tongue squamous-cell carcinoma utilizing the polymerase chain reaction. *Int J Cancer* **50**: 167–171

Shoji Y, Saegusa M, Takano Y, Ohbu M and Okajasu I (1996) Correlation of apoptosis with tumour cell differentiation, progression, and HPV infection in cervical carcinoma. *J Clin Pathol* **49**: 134–138

Smith ML, Chen IT, Zhan Q, O'Connor PM and Fornace AJJ (1995) Involvement of the p53 tumour suppressor in repair of u.v.-type DNA damage. *Oncogene* **10**: 1053–1059

Snijders PJF, van den Brule AJC, Meijer CJLM and Walboomers JMM (1994) Papillomaviruses and cancer of the upper digestive and respiratory tracts. *Curr Topics Microbiol Immunol* **186**: 177–198

Tatebe S, Ishida M, Kasagi N, Tsujitani S, Kaibara N and Ito H (1996) Apoptosis occurs more frequently in metastatic foci than in primary lesions of human colorectal carcinomas: Analysis by terminal-deoxynucleotidyl transferase mediated dUTP biotin nick end labeling. *Int J Cancer* **65**: 173–177

Törmänen U, Eerola A, Rainio P, Vähäkangas K, Soini Y, Sormunen R, Bloigu R, Lehto V and Pääkkö P (1995) Enhanced apoptosis predicts shortened survival in non-small cell lung carcinoma. *Cancer Res* **55**: 5595–5602

Weinberg RA (1991) Tumour suppressor genes. *Science* **254**: 1138–1146

Wyllie AH, Morris RG, Smith AL and Dunlop D (1984) Chromatin cleavage in apoptosis: association with condensed chromatin morphology and dependence on macromolecular synthesis. *J Pathol* **142**: 67–77

Yoshikawa H, Kawana T, Kitagawa K, Mizuno M, Yoshikura H and Iwamoto A (1991) Detection and typing of multiple genital human papillomaviruses by DNA amplification with consensus primers. *Jpn J Cancer Res* **82**: 524–531

zur Hausen H (1994) Molecular pathogenesis of cancer of the cervix and its causation by specific human papillomavirus types. *Curr Topics Microbiol Immunol* **186**: 131–156

© Cancer Research Campaign 1998